# International Criteria for Reporting Study Quality for Sudden Cardiac Arrest/Death Tool

**DOI:** 10.1161/JAHA.123.033723

**Published:** 2024-05-23

**Authors:** Jamie J. Edwards, Claire Compton, Nikhil Chatrath, Bradley J. Petek, Aaron Baggish, Mats Börjesson, Eugene Chung, Domenico Corrado, Jonathan A. Drezner, Sabiha Gati, Belinda Gray, Jonathan Kim, Andre La Gerche, Aneil Malhotra, Eloi Marijon, Michael Papadakis, Antonio Pelliccia, Dermot Phelan, Chris Semsarian, Sanjay Sharma, Rajan Sharma, Jamie M. O'Driscoll, Kimberly G. Harmon

**Affiliations:** ^1^ School of Psychology and Life Sciences Canterbury Christ Church University Kent UK; ^2^ Department of Cardiology South Tees Hospitals National Health Service Foundation Trust, The James Cook University Hospital Middlesbrough UK; ^3^ Cardiology Clinical Academic Group, St George’s University of London London UK; ^4^ Division of Cardiology Massachusetts General Hospital Boston MA; ^5^ Cardiovascular Performance Program Massachusetts General Hospital Boston MA; ^6^ Center for Lifestyle Intervention, Medicine, Geriatrics and Emergency Department Sahlgrenska University Hospital Göteborg Sweden; ^7^ Department of Molecular and Clinical Medicine Institute of Medicine, Sahlgrenska Academy, University of Gothenburg Göteborg Sweden; ^8^ University of Michigan, West Michigan Program, Cardiac Electrophysiology Service, Sports Cardiology Clinic, Michigan Medicine Ann Arbor MI; ^9^ Department of Cardiac, Thoracic, Vascular Sciences and Public Health University of Padova Padova Italy; ^10^ Department of Family Medicine, Center for Sports Cardiology University of Washington Seattle WA; ^11^ National Heart and Lung Institute, Imperial College London London UK; ^12^ Department of Cardiology Royal Brompton Hospital London London UK; ^13^ Agnes Ginges Centre for Molecular Cardiology Centenary Institute New South Wales Australia; ^14^ Faculty of Health and Medical Sciences University of Sydney New South Wales Australia; ^15^ Department of Cardiology Royal Prince Alfred Hospital New South Wales Australia; ^16^ Emory School of Medicine Emory Clinical Cardiovascular Research Institute Atlanta GA; ^17^ Clinical Research Domain Baker Heart and Diabetes Institute, Alfred Centre Melbourne Victoria Australia; ^18^ National Centre for Sports Cardiology Fitzroy Victoria Australia; ^19^ Cardiology Department St Vincent’s Hospital Melbourne Fitzroy Victoria Australia; ^20^ Institute of Sport Manchester Metropolitan University and Manchester University NHS Foundation Trust Manchester UK; ^21^ Paris Cardiovascular Research Center INSERM U970, Hôpital Européen Georges Pompidou Paris France; ^22^ Institute of Sports Medicine and Science Rome Italy; ^23^ Sports Cardiology Center, Sanger Heart and Vascular Institute, Atrium Health Charlotte NC; ^24^ Department of Cardiology St George’s University Hospitals NHS Foundation Trust Tooting, London UK

**Keywords:** athletes’ heart, SCA/D, sports cardiology, sudden cardiac death, Quality and Outcomes

## Abstract

**Background:**

Studies reporting on the incidence of sudden cardiac arrest and/or death (SCA/D) in athletes commonly lack methodological and reporting rigor, which has implications for screening and preventative policy in sport. To date, there are no tools designed for assessing study quality in studies investigating the incidence of SCA/D in athletes.

**Methods and Results:**

The International Criteria for Reporting Study Quality for Sudden Cardiac Arrest/Death tool (IQ‐SCA/D) was developed following a Delphi process. Sixteen international experts in sports cardiology were identified and invited. Experts voted on each domain with subsequent moderated discussion for successive rounds until consensus was reached for a final tool. Interobserver agreement between a novice, intermediate, and expert observer was then assessed from the scoring of 22 relevant studies using weighted and unweighted κ analyses. The final IQ‐SCA/D tool comprises 8 domains with a summated score of a possible 22. Studies are categorized as low, intermediate, and high quality with summated IQ‐SCA/D scores of ≤11, 12 to 16, and ≥17, respectively. Interrater agreement was “substantial” between all 3 observers for summated IQ‐SCA/D scores and study categorization.

**Conclusions:**

The IQ‐SCA/D is an expert consensus tool for assessing the study quality of research reporting the incidence of SCA/D in athletes. This tool may be used to assist researchers, reviewers, journal editors, and readers in contextualizing the methodological quality of different studies with varying athlete SCA/D incidence estimates. Importantly, the IQ‐SCA/D also provides an expert‐informed framework to support and guide appropriate design and reporting practices in future SCA/D incidence trials.

**Nonstandard Abbreviations and Acronyms**
IQ‐SCA/D: International Criteria for Reporting Study Quality for Sudden Cardiac Arrest/Death
SCA/D: sudden cardiac arrest/death

**Clinical Perspective**

**What Is New?**

- This expert consensus process presents the development of the International Criteria for Reporting Study Quality for Sudden Cardiac Arrest/Death tool.
- The International Criteria for Reporting Study Quality for Sudden Cardiac Arrest/Death tool is a new tool designed for assessing study quality in incidence studies of sudden cardiac arrest/death in athletes, providing an expert‐informed framework to support and guide appropriate design and reporting practices in future trials.

**What Are the Clinical Implications?**

- This tool may assist clinicians, researchers, reviewers, journal editors, and readers in contextualizing the methodological quality of past and future studies with varying incidence estimates, ultimately leading to an improved understanding of sudden cardiac arrest/death frequency in athletes.

Sudden cardiac arrest/death (SCA/D) in athletes is a devastating event with widespread implications.^1^ Although SCA/D is often characterized as infrequent,^2^, ^3^, ^4^ a lack of methodological and reporting standardization has resulted in conflicting and far‐ranging estimates of SCA/D events in athletes without the appropriate population and methodological homogeneity across different studies to establish the clear moderators driving these differences in estimates. Studies extensively vary in design (prospective versus retrospective), numerator and denominator calculation, inclusivity of sudden cardiac arrest cases, appropriateness of the reporting window (sports‐related versus anytime SCA/D), subgroup data reporting practices (sport and ethnicity‐specific incidence), and important confounders, such as pooling data from different age groups and sexes.

Establishing accurate, context‐specific incidence estimates is imperative to understanding the appropriateness of preplanned screening initiatives and preventative policy in sport, as well as the consideration of defibrillator placement and emergency action planning for on‐field SCA/D events. Therefore, estimate inaccuracy carries widespread implications. To date, there are no tools specifically designed for assessing study quality in studies investigating the incidence of SCA/D in athletes. Indeed, previous systematic reviews in this area have resorted to using customized versions or tools that may not accurately reflect risk of bias.^5^, ^6^ Well‐designed study assessment tools can provide insight into the potential accuracy or context‐specific interpretation of an incidence estimate. Furthermore, the domains of a relevant assessment tool can provide a comprehensive framework for appropriate design and data reporting practices for future trials.

The objective of this international expert consensus was to develop and validate the interobserver reliability of a novel tool designed for assessing methodological and reporting quality of incidence studies of SCA/D in athletes. A Delphi process method was preplanned to support the development of the International Criteria for Reporting Study Quality for Sudden Cardiac Arrest/Death tool (IQ‐SCA/D).

## Methods

The authors declare that all supporting data are available within the article (and its online supplementary files). Institutional review board approval and informed consent were not required for this study.

The Delphi process followed in the creation of the IQ‐SCA/D can be visualized in the Figure.^7^

**Figure .**
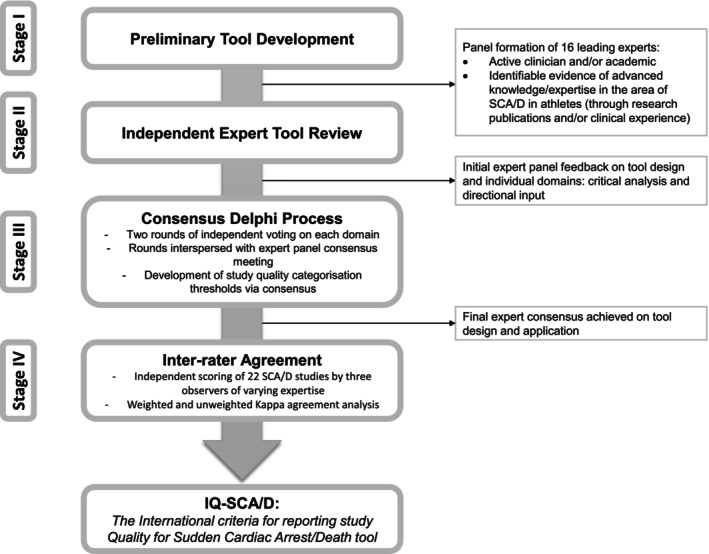
The Delphi process followed in the creation of the International Criteria for Reporting Study Quality for Sudden Cardiac Arrest/Death (IQ‐SCA/D) tool. SCA/D indicates sudden cardiac arrest/death.

### Aims of the Tool

The primary aims of this tool are 2‐fold: first, to provide a reliable study quality assessment index of incidence estimate trials of SCA/D in athletes; and second, to provide an expert‐informed framework to support and guide appropriate design and reporting practices in future SCA/D incidence trials. The rating scale of this tool is specifically designed to provide the highest quality scoring to those studies with the most accurate incidence estimates of SCA/D in athletes.

### Stage 1

Stage 1 involved the development of a preliminary draft tool by the primary authors (J.E., J.'O.D., and K.H.). This preliminary draft tool was produced as an adaptation to the most frequently used prevalence tools, including the Joanna‐Briggs Institute critical appraisal checklist,^5^, ^6^, ^8^, ^9^ with consideration of the frequently cited limitations identified when applying these tools in SCA/D incidence studies. This draft tool can be seen in Data S1. Following this, a list of global experts and key opinion leaders was subjectively compiled, and each individually contacted to assess interest. Potential experts were considered if they met the following criteria:

- Active clinician or academic
- Identifiable evidence of advanced knowledge/expertise in the area of SCA/D in athletes (eg, through research publications, clinical experience)
- Time availability to engage in the Delphi process

All participants accepted the invitation, with a resulting expert panel of 16 international expert academics/clinicians in sports cardiology.

### Stage 2

Stage 2 presented all experts with a copy of the preliminary draft tool in which they were asked to independently review and provide comprehensive written feedback with critical analysis and directional input on each domain. The primary authors then adjusted the tool through implementation of the written feedback ready for initialization of the Delphi process.

### Stage 3: Delphi Process

Stage 3 introduced the Delphi process, consisting of 2 rounds of anonymous panel voting on the newly adjusted expert‐informed tool. All experts received a Microsoft Forms document (Data S2) in which they provided anonymous votes on each domain of the tool.

Each domain had the following 3 voting options:

- A. “Yes, I support the category and scoring as is”
- B. “I would like to discuss the category or the scoring further”
- C. “No, I do not support the inclusion of the category”

If ≥80% of the expert respondents voted “yes,” then the domain was accepted without further discussion. If ≥80% of the respondents voted “no,” then the domain was rejected without further discussion. If neither of the above conditions was met, the domain was opened to further moderated discussion in the form of a video call meeting until the 80% yes threshold could be reached. If disagreement had persisted, a primary dissenter or group of dissenters would have been asked to write a short paragraph explaining their position to be published with the article.

In the first Delphi round, 2 domains (Data S3) did not meet either acceptance or rejection criteria, and therefore these domains were discussed in a video call meeting where all experts (both dissenters and nondissenters) provided input. Common discussion points surrounded optimization of the written descriptions and point weightings for each item listed within a domain. Following the moderated discussion and subsequent tool adjustments, the second Delphi round was methodologically identical to round 1, with a repeat of the voting protocol, but only on those domains not accepted in voting round 1. The second Delphi round observed that the acceptance criteria were met for both domains. As such, the resulting tool therefore reflects the consensus recommendations made by the panel of experts. Study quality (low, intermediate, and high quality) categorization thresholds were also developed through expert consensus.

### Stage 4: Interobserver Reliability Assessment

Three observers of varying expertise (novice, intermediate, and expert observer) in the field of SCA/D in athletes were identified as suitable to perform independent study assessments using the IQ‐SCA/D tool. The novice, intermediate, and expert observers were defined according to their level of engagement with the relevant athlete and SCA/D literature. The novice observer had no research experience in the area, the intermediate had some experience and knowledge in supporting previous work, and the expert has led the development of multiple published studies in the subject area. The observers were separate from the experts who participated in the Delphi process. Twenty‐two published SCA/D incidence trials were identified from a local database and distributed to the observers alongside the tool.

Agreement between the observers was statistically assessed using weighted κ analyses to take into account the seriousness of the disagreement between observers.^10^ Unweighted Cohen κ was applied to domain 7 as the only domain to include 2 ordinal categories. Fleiss κ was also used to assess study quality categorization across the multiple observers in addition to the paired observer analysis. This statistical approach has been applied in the reliability testing and validation process of several previous assessment tools.^9^, ^11^, ^12^ Agreement between each observer (1 versus 2, 2 versus 3, and 1 versus 3) was assessed for each individual domain, the total summated study scores, and study quality categorization. The level of agreement can be described according to Landis and Koch,^13^ as follows: >0.81 “almost perfect” (a); 0.61 to 0.80 “substantial” (b); 0.41 to 0.60 “moderate” (c); 0.21 to 0.40 “fair” (d); 0.00 to 0.20 “slight” and 0.00 “poor” (e). All analyses were performed using SPSS, version 28.0.1, and results were considered statistically significant with a *P*<0.05.

## Results

The IQ‐SCA/D tool is a specialized quality assessment tool for studies reporting incidence of SCA/D in athletes. The final tool contains 8 domains of varying weighting, with a total possible score of 22. A concise summary of the IQ‐SCA/D can be found in Table 1.^14^, ^15^, ^16^, ^17^, ^18^, ^19^, ^20^, ^21^, ^22^, ^23^, ^24^, ^25^, ^26^, ^27^, ^28^ Table 2, ^2^, ^24^, ^28^, ^29^, ^30^, ^31^, ^32^, ^33^, ^34^, ^35^, ^36^, ^37^, ^38^, ^39^, ^40^, ^41^, ^42^, ^43^, ^44^, ^45^, ^46^, ^47^ provides study characteristics and quality categorization of the 22 scored SCA/D studies.

**Table 1:** The IQ‐SCA/D

Domain	Context	Scoring
Domain 1: study design score	Study design is an important feature related to quality, and there are generally accepted levels of evidence ranging from systematic reviews to expert opinion.^14^, ^15^ This category provides scoring based on whether the study design is prospective or retrospective. Examples of search strategies that would fall into each category are provided	3 Points: prospective (ie, prospective active monitoring and collection of new SCA/D cases with defined search strategy) 2 Points: prospective and retrospective (ie, uses both prospective monitoring for new cases and retrospective review of previous cases) 1 Point: retrospective (ie, retrospective search of media reports; retrospective application of a search strategy; retrospective review of autopsy records) 0 Points: retrospective survey (ie, survey to report past cases of SCA/D)
Domain 2: numerator/strength of case identification	Identifying cases of SCA/D is difficult and often limited by the lack of mandatory reporting systems and ill‐defined athlete populations.^17^ The method used is important and correlates with the likelihood of capturing all cases.^18^, ^19^ Research^27^ has shown the variable sensitivity of different case identification approaches (eg, media reports and insurance claims identifying only 62% and 19% of cases, respectively), highlighting the importance of mandatory systems/the use of multiple case identification sources. The accuracy of case identification is 1 of the more important aspects of studies on SCA/D and therefore weighted more heavily with 5 points. This category attempts to rate both the accuracy of case identification and the ability to identify athletes specifically. Examples of methods are provided for each category, but some studies may fit into >1 category. Points should be awarded on the basis of the overall likelihood of the identification of all SCA/D in athletes	5 Points: mandatory reporting system for all cases of SCA/D in athletes. The mandatory reporting system does not have to be athlete specific; however, if there is mandatory reporting of a larger population, there should be a reliable way to identify the precise number of competitive athlete cases 4 Points: use of multiple search strategies that increase the likelihood of case identification (≥2): (post‐Internet [after 2005] media reports, other databases or registries, death certificate records review, and other nonmandatory reporting methods) 3 Points: media reports post‐Internet (after 2005) in an athlete population likely to be documented (professional, collegiate athletes) but without the use of other case identification methods 2 Points: media reports in a population that is unlikely to be well documented (ie, middle‐school, high school, and recreational/noncompetitive athletes) 1 Point: reliant on recall (ie, survey) mandatory reporting (death certificate) with unclear designation of athlete status (ie, population database where it is difficult to accurately identify which cases are in competitive athletes) 0 Points: methods that are unlikely to identify the majority of SCA/D in athletes (media reports pre‐Internet [2005 or before] in isolation, catastrophic insurance claims, limited sources [ie, newspapers] unlikely to identify all cases, review of autopsy reports where not all SCD cases have autopsies, and does not state how cases were identified)
Domain 3: denominator	The denominator of an incidence proportion is the number of people at the start of an observation period. Studies of SCA/D should clearly define what population they are studying and how the group is determined. Many studies estimate participation (ie, “there are about 8 000 000 high school athletes^20^, ^21^), which can result in either overestimation or underestimation of risk. The denominator should define the number of individual athletes participating during a defined observation period.^22^, ^23^ Examples of different strategies are provided	3 Points: precisely defined (ie, registered athletes in a database, known number of participants in a league) 2 Points: defined population but numbers may not be exact (ie, estimates of the number of athletes in a league) 1 Point: use of a multiplier with a precisely defined population (multipliers are sometimes used to account for multisport athletes in a known athletic population) 0 Points: estimate (ie, estimated number of participants per year, general population statistics, based on reported physical activity surveys)
Domain 4: all vs Sports Related Sudden Cardiac Death	Sports/exercise‐related SCA/D vs SCA/D that occurs at any time of the day are different, but this is often not recognized. Sports/exercise‐related SCA/D is typically defined as death that occurs during or within an hour of exercise and is a subset of all SCA/D in athletes. Sports/exercise‐related SCA/D is an important metric to consider when event planning or creating emergency action plans; however, it should not be conflated with SCA/D that occurs at any time, inclusive of any activity, rest, and sleep	2 Points: all SCA/D at any time regardless of activity or physical exertion 1 Point: only sports/exercise‐related SCA/D (occurring within an hour of sports or exercise) or SCA/D that occurs during a specific time portion of the day (ie, school or work day) 0 Points: unclear whether included cases are all SCA/D or sports/exercise‐related SCA/D
Domain 5: SCA/D vs SCD only	Most studies of SCA/D in athletes include only SCD in their analysis; however, the inclusion of SCA with survival is important to understand the scope of the problem. Studies including both SCA and SCD show that as many as 50% athletes who experience SCA are resuscitated.^24^, ^28^	3 Points: inclusive of both SCA with survival and SCD with reliable reporting mechanisms for both SCA and SCD (ie, prospective study with mandatory reporting of both SCA and SCD) 2 Points: inclusive of both SCA and SCD but mechanisms for identification (of either SCA or SCD) may not be robust 1 Point: clearly defines whether study includes only SCA or SCD 0 Points: does not define inclusion criteria
Domain 6: age range	Grouping wide age ranges together can lead to inaccurate estimates of the incidence of SCA/D. Population‐based studies demonstrate a peak in SCA/D in those <1 year of age followed by a relatively low rate of SCA/D that increases again around age 15 years before rising precipitously at age 25 years because of the increasing contribution of coronary artery disease.^3^, ^18^, ^25^ In those <25 years old, the primary causes of SCA/D are inherited structural and electrical cardiac diseases.^3^ Many studies of SCA/D group wide swaths of ages (ie, 12–40 years) with widely varying incidence rates and causes of SCA/D calculated together (see Table 2). For an accurate estimation of the incidence rate, it is important that the age grouping reflects a similar risk of SCA/D in that group	2 Points: age groups are generally aligned with risk (ie, high school, college, 12–14, >14–18, >18–25, >25–35, >35 years; or child, adolescent, young adult, adult) 1 Point: age groups include varying risk but do not include overlapping primary causes (ie, age 12–25 years) 0 Points: wide age range with varying risk or ages grouped with different predominant causes (inherited disorders vs coronary artery disease) combined (ie, 12–40 years)
Domain 7: sex‐specific rates	Studies of SCA/D in athletes and nonathletes alike have consistently shown that male individuals have a higher rate of SCA/D than female individuals. In general, male individuals have 3–4 times higher rates of SCA/D. Combining both male and female individuals in the same groups artificially lowers the risk for male individuals and increases the risk for female individuals.^3^, ^18^, ^25^ There need to be sex‐specific numbers available for both the numerator and denominator so that an incidence rate can be calculated for both sexes	2 Points: sex‐specific groups and incidence calculations possible (including if study is only 1 sex [ie, male]) 0 Points: it is not possible for sex‐specific rates to be calculated
Domain 8: subgroup reporting	There may be important subgroup risks, such as sport or ethnicity.^3^, ^23^, ^26^ There needs to be ethnicity or sport‐specific numbers available for both the numerator and the denominator	2 Points: sport and racial and ethnic incidence rates are reported or can be calculated (including if study was only done in 1 sport) 1 Point: incidence rates are reported/can be calculated for sport (including if study was only done in 1 sport) but not race and ethnicity or race and ethnicity but not sport 0 Points: there is no subgroup data reporting

IQ‐SCA/D indicates International Criteria for Reporting Study Quality for Sudden Cardiac Arrest/Death; SCA, sudden cardiac arrest; SCA/D, sudden cardiac arrest/death; and SCD, sudden cardiac death.

**Table 2:** SCA/D Incidence Studies Scored for Interobserver Reliability

Study	Study design and population	Case identification (numerator)	Population definition (denominator)	Sports‐related SCD or all SCD?	SCD or all SCA/D?	Study years	Age range, y; number of cases	Annual incidence	Study quality
Bohm 2016^29^	Prospective cohort; sports‐related SCD in all people in Germany	Voluntary reporting to German National Registry, web‐based media search, regional institutes	Physical activity estimated from the German Health Update study and extrapolated to population data from the German Federal Statistical Office	Sports‐related SCD	SCD	2012–2014	10–79 N=144	*Sports participants* 1:1200000	Low
Chatard 2018^30^	Prospective, Pacific Island athletes who were screened	Prospectively followed	Defined cohort of 1450 athletes		SCD	2012–2015	10–40 N=3	*Pacific Island athletes* 1:2416	Low
Corrado 2003^31^	Prospective cohort; athletes and nonathletes in the Veneto Region of Italy	Mandatory reporting of sudden death	Registered athletes in the Sports Medicine Database of the Veneto Region of Italy and the Italian Census Bureau	All	SCD	1979–1999	12–35 N=51 12–35 N=208	*Athletes* Overall 1:47000 Male 1:41000 Female 1:93000 Nonathletes Overall 1:143 000	High
Corrado 2006^32^	Prospective cohort; athletes and nonathletes in the Veneto Region of Italy	Mandatory reporting of sudden death	Registered athletes in the Sports Medicine Database of the Veneto Region of Italy and the Italian Census Bureau	All	SCD	1979–2004	12–35 N=55 12–35 N=265	*Athletes* Overall: 1:53000 Nonathletes Overall: 1:127000	High
Drezner 2005^24^	Retrospective survey; college athletes	Survey of NCAA Division I institutions (244/326 responded)	Reported number of athletes	All	SCD	__	N=5	*College* Overall 1:67 000	Low
Drezner 2009^28^	Cross‐sectional survey; high school athletes	Survey of 1710 high schools with AEDs	Reported number of student athletes	All cases occurring on campus	SCA+SCD	2006–2007	14–17 N=14	*High school* 1:23 000 (SCA+SCD) 1:64 000 (SCD)	Low
Drezner 2014^33^	Retrospective cohort; Minnesota high school athletes	Public media reports	Minnesota State High School League statistics (sum of unduplicated athletes 2003–2004 through 2011–2012 school years)	All	SCA+SCD	2003–2012	14–18 N=13	*High school* Overall 1:71 000 Female 0 Male, basketball 1:21 000	Intermediate
Grani 2016^34^	Retrospective; sports‐related SCD in all people in German‐speaking Switzerland	Forensic reports	Physical activity estimated from survey on sports participation by the Swiss Federal Office of Sports	Sports‐related SCD	SCD	1999–2010	10–39 N=69	*Sports participants* Competitive: 1:90 000 Recreational: 1:192 000	Low
Harmon 2015^35^	Retrospective cohort; college athletes	Parent Heart Watch database, NCAA Resolutions list, catastrophic insurance claims	Participation data from the NCAA	All	SCD	2003–2013	17–26 N=79	*College* Overall 1:53 000 Male 1:38 000 Female 1:122 000 Black 1:21 000 White 1:68 000 Football 1:36 000 Male, soccer 1:24 000 Male, Black 1:16 000 Male, basketball 1:9000 Male, Black, basketball 1:5300 Male, division I basketball 1:5200	High
Harmon 2016^36^	Retrospective cohort, US high school athletes	Media reports	National Federation of State High School Associations participation statistics	All	SCA/SCD	2007–2013	14–18 N=104	*High school* Overall 1:67 000 Male 1:4500 Female 1:237 000 Male, basketball 1:37 000	Intermediate
Holst 2010^37^	Retrospective cohort; athletes and general population in Denmark	Review of death certificates, Cause of Death Registry, and National Patient Registry in Denmark	Interview data of people aged 16–35 y from the National Danish Health and Morbidity Study	Sports‐related SCD in athletes vs all SCD in the general population	SCD	2000–2006	12–35 N=15 12–35 N=428	*Athletes* 1:83000 *General population* 1:27 000	Low
Malhotra 2018^38^	Prospective	Followed from time of screen to 2016	Defined cohort of 11 168 elite soccer athletes	All	SCD	1996–2016	15–17 N=8	*Elite male soccer athletes* 1:14 794	Intermediate
Marijon 2011^39^	Prospective cohort; general population in France	Data from emergency medical system	General population statistics, data from the Minister of Health and Sport to estimate young competitive athlete population	Sports‐related SCA or SCD with moderate or vigorous exercise	SCA+SCD	2005–2010	10–75 N=820 10–35 N=50	*General population* *1:217 000* *Young competitive athlete* *1:102 000* *Young noncompetitive athlete* *1:455 000*	Intermediate
Maron 2009^40^	Retrospective cohort; amateur and competitive athletes	US Registry for Sudden Death in Athletes	An estimated 10.7 million participants per year ≤39 y of age in all organized amateur and competitive sports	All	SCA+SCD	1980–2006	8–39 N=1046	*Athletes* 1:164 000	Low
Maron 2013^41^	Retrospective cohort; Minnesota high school athletes	US Registry for Sudden Death in Athletes	Minnesota State High School League statistics (estimated using conversion factor of 2.3 to account for multisport athletes)	All	SCD	1986–2011	12–18 N=13	*High school* Overall 1:150 000 Male 1:83 000 Female 0	Intermediate
Maron 2016^42^	Retrospective cohort	Records of the Medical Examiner	Data from the Minnesota Department of Education, National Center for Education Statistics, and the Minnesota State High School League for Hennepin County, Minnesota	All	SCD	2000–2014	14–23 N=27	*Nonathlete* 1:39 000 *Athlete* 1:121 000	Low
Peterson 2021^2^	Prospective	National Center for Catastrophic Sports Injury Research	Has defined cohort for high school and college athletes	All	SCA/SCD	2014–20181:	14–18 N=204 18–24 N=39	*High school* Overall 1:66 000 Male 1:4400 Female 1:204000 Male ice hockey 1:24 000 Male basketball 1:40 000 *College* Overall 1:51 000 Male 1:35 000 Female 1:123 000 Black Male 1:18 000 White Male 1:39 000 Black Male basketball 1:4800 White Male basketball 1:15 000 Black football 1:28 000 White football 1:20 000	High
Risgaard 2014^43^	Retrospective cohort; competitive and noncompetitive athletes in Denmark	Review of death certificates and the Danish National Patient Registry	Competitive and noncompetitive athlete populations in Denmark estimated based on survey data from the Danish National Institute of Public Health	Sports‐related SCD in competitive vs noncompetitive athletes	SCD	2007–2009	12–35 N=44	*Competitive athlete* 1:213 000 *Noncompetitive athlete* 1:233 000	Low
Roberts 2013^44^	Retrospective cohort	Catastrophic insurance records	Minnesota State High School League statistics (sum of unduplicated athletes 1993/1994 through 2011–2012 academic years)	Sports‐related SCD in high school practice or games	SCD	1993/1994–2011–12	12–19 N=4	*High school athlete* 1:417 000	Low
Steinvil 2011^45^	Retrospective cohort; athletes in Israel	Retrospective review of 2 Israeli newspapers	Competitive athletes registered in the Israel Sport Authority in 2009; extrapolated these data for prior 24 y based on the growth of the Israeli population (aged 10–40 y) from the Central Bureau of Statistics; allowed for a presumed doubling of the sporting population over 24 y	All	SCD	1985–2009	12–44 N=24	*Athletes* 1:38 000	Low
Toresdahl 2014^46^	Prospective observational; high school students and student‐athletes	2149 high schools monitored for SCA events on school campus	Reported number of students and student‐athletes	All cases occurring on school campus	SCA+SCD	2009–2011	14–18 N=44	*Student‐athlete* Overall 1:88 000 Male 1:58 000 Female 1:323 000 *Student nonathlete* Overall 1:326 000 Male 1:286 000 Female 1:357 000	High
Van Camp 1996^47^	Retrospective cohort; high school and college athletes	National Center for Catastrophic Sports Injury Research and media reports	Data from NCAA, NFHS, NAIA, and NJCAA, added together with conversion factor (1.9 for high school and 1.2 for college) used to account for multisport athletes “based on discussions with representatives from the national organizations”	Sports‐related	SCD	1983–1993	13–24 N=160	*College*±*high school* Overall 1:188 000 Male 1:134 000 Female 1:752 000 *High school* Overall 1:213 000 Male 1:152 000 Female 1:861 000 *College* Overall 1:94 000 Male 1:69 000 Female 1:356 000	Low

NAIA indicates National Association of Intercollegiate Athletics; NCAA, National Collegiate Athletics Association; NFHS, National Federation of State High School Associations; NJCAA, National Junior College Athletic Association; SCA, sudden cardiac arrest; SCA/D, sudden cardiac arrest/death; and SCD, sudden cardiac death.

### Domain 1: Study Design

Study design is an important feature related to quality, and there are generally accepted levels of evidence ranging from systematic reviews to expert opinion. This category provides scoring based on whether the study design is prospective or retrospective. Examples of search strategies that would fall into each category are provided.

### Domain 2: Numerator/Strength of Case Identification

Identifying cases of SCA/D is difficult and often limited by the lack of mandatory reporting systems and ill‐defined athlete populations. The method used is important and correlates with the likelihood of capturing all cases. The accuracy of case identification is one of the more important aspects of studies on SCA/D and therefore weighted more heavily with 5 points. This category attempts to rate both the accuracy of case identification and the ability to identify athletes specifically. Examples of methods are provided for each category, but some studies may fit into >1 category. Points should be awarded on the basis of the overall likelihood of the identification of all SCA/D in athletes.

### Domain 3: Denominator

The denominator of an incidence proportion is the number of people at the start of an observation period. Studies of SCA/D should clearly define what population they are studying and how the group is determined. Many studies estimate participation (ie, “there are ≈8 000 000 high school athletes”), which can result in either overestimation or underestimation of risk. The denominator should define the number of individual athletes participating during a defined observation period. Examples of different strategies are provided.

### Domain 4: All Cases Versus Sports/Exercise‐Related Cases

Sports/exercise‐related SCA/D versus SCA/D that occurs at any time of the day are different, but this is often not recognized. Sports/exercise‐related SCA/D is typically defined as death that occurs during or within an hour of exercise and is a subset of all SCA/D in athletes. Sports/exercise‐related SCA/D is an important metric to consider when event planning or creating emergency action plans; however, it should not be conflated with SCA/D that occurs at any time, inclusive of any activity, rest, and sleep.

### Domain 5: SCA/D Versus Sudden Cardiac Death Only

Most studies of SCA/D in athletes include only sudden cardiac death in their analysis; however, the inclusion of sudden cardiac arrest with survival is important to understand the scope of the problem. Studies including both sudden cardiac arrest and sudden cardiac death show that as many as 50% of athletes who experience sudden cardiac arrest are successfully resuscitated.

### Domain 6: Age Range

Grouping wide age ranges together can lead to inaccurate estimates of the incidence of SCA/D. Population‐based studies demonstrate a peak in SCA/D in those aged <1 year followed by a relatively low rate of SCA/D that increases again around age 15 years before rising precipitously at age 25 years because of the increasing contribution of coronary artery disease. In those aged <25 years, the primary causes of SCA/D are inherited structural and electrical cardiac diseases. Many studies of SCA/D group wide swaths of ages (ie, 12–40 years) with widely varying incidence rates and causes of SCA/D calculated together. For an accurate estimation of the incidence rate, it is important that the age grouping reflects a similar risk of SCA/D in that group.

### Domain 7: Sex‐Specific Rates

Studies of SCA/D in athletes and nonathletes alike have consistently shown that male individuals have a higher rate of SCA/D than female individuals. In general, male individuals have 3 to 4 times higher rates of SCA/D. Combining both male and female individuals in the same groups artificially lowers the risk for male individuals and increases the risk for female individuals. As such, there is a need for sex‐specific data available for both the numerator and denominator so that an accurate incidence rate can be calculated for both sexes.

### Domain 8: Subgroup Reporting

There may be important subgroup risks, such as sport or race and ethnicity. There needs to be sport‐specific or race and ethnicity numbers available for both the numerator and the denominator.

### Interobserver Agreement

Interobserver agreement for each domain, the total summative scoring, and the study quality categorization can be seen in Table 3. Domain agreement ranged from “fair” to “almost perfect,” whereas agreement for both total summated scores (observer 1 versus 2: 0.610k±0.06, 2 versus 3: 0.660k±0.06, 1 versus 3: 0.616k±0.08 (k = Kappa)) and the study quality categorization (observer 1 versus 2: 0.753k±0.11, 2 versus 3: 0.763k±0.11, 1 versus 3: 0.641k±0.14) was consistently “substantial” across all observers. This was further supported by the substantial study quality categorization from the Fleiss κ analysis (0.655±0.093 [95% CI, 0.473–0.837]). The substantial agreement in total scoring and quality categorization across the observers supported the categorization of studies as low, intermediate, or high quality with summated IQ‐SCA/D scores of ≤11, 12 to 16, and ≥17, respectively.

**Table 3:** Interobserver Agreement Analysis Results

IQ‐SCA/D domain	Observer 1 vs 2	Observer 2 vs 3	Observer 1 vs 3
Domain 1: study design	0.770 (SE=0.089, 95% CI=0.596–0.944)	0.911 (SE=0.060, 95% CI=0.793–1.028)	0.693 (SE=0.093, 95% CI=0.511–0.875)
Domain 2: numerator/strength of case identification	0.780 (SE=0.107, 95% CI=0.570–0.990)	0.779 (SE=0.120, 95% CI=0.544–1.015)	0.813 (SE=0.110, 95% CI=0.597–1.029)
Domain 3: denominator	0.526 (SE=0.125, 95% CI=0.281–0.772)	0.588 (SE=0.136, 95% CI=0.321–0.855)	0.447 (SE=0.124, 95% CI=0.204–0.689)
Domain 4: all cases vs sports‐related cases	0.298 (SE=0.186, 95% CI=−0.067‐0.662)	0.548 (SE=0.187, 95% CI=0.182–0.914)	0.403 (SE=0.190, 95% CI=0.032–0.775)
Domain 5: SCA/D vs SCD	0.345 (SE=0.131, 95% CI=0.089–0.602)	0.498 (SE=0.153, 95% CI=0.197–0.798)	0.575 (SE=0.098, 95% CI=0.382–0.768)
Domain 6: age range	0.494 (SE=0.134, 95% CI=0.232–0.756)	0.745 (SE=0.124, 95% CI=0.503–0.988)	0.639 (SE=0.116, 95% CI=0.412–0.867)
Domain 7: sex‐specific rates	0.648 (SE=0.149, 95% CI=0.356–0.940)	0.624 (SE=0.170, 95% CI=0.292–0.956)	0.472 (SE=0.173, 95% CI=0.133–0.811)
Domain 8: subgroup reporting	0.320 (SE=0.169, 95% CI=−0.011–0.650)	0.644 (SE=0.122, 95% CI=0.404–0.883)	0.482 (SE=0.173, 95% CI=0.143–0.820)
Total summative scores	0.610 (SE=0.057, 95% CI=0.499–0.721)	0.660 (SE=0.060, 95% CI=0.541–0.779)	0.616 (SE=0.083, 95% CI=0.453–0.778)
Quality category agreement	0.753 (SE=0.110, 95% CI=0.537–0.969)	0.763 (SE=0.112, 95% CI=0.543–0.983)	0.641 (SE=0.138, 95% CI=0.371–0.912)
Quality category Fleiss κ	0.655 (SE=0.093, 95% CI=0.473–0.837)		

Data reported as κ, SE, and 95% CI. Observers 1, 2, and 3 represent the expert, intermediate, and novice observers, respectively. IQ‐SCA/D indicates International Criteria for Reporting Study Quality for Sudden Cardiac Arrest/Death; SCA/D, sudden cardiac arrest/death; and SCD, sudden cardiac death.

Observers 1 and 2 achieved substantial agreement for 3 of 8 domains, moderate agreement for 2 of 8 domains, and fair agreement for 3 of 8 domains. Observers 2 and 3 achieved almost perfect agreement for 1 of 8 domains, substantial agreement for 4 of 8 domains, and moderate agreement for 3 of 8 domains. Observers 1 and 3 achieved almost perfect agreement for 1 of 8 domains, substantial agreement for 2 of 8 domains, and moderate agreement for 5 of 8 domains. In practice, this tool should be applied by 2 observers independently, and disagreements should be resolved with the help of a third reviewer.

## Discussion

This international expert consensus presents the development and interobserver reliability of the IQ‐SCA/D, a novel tool designed for assessing study quality in studies investigating the incidence of SCA/D in athletes. The IQ‐SCA/D provides an expert‐informed framework to support and guide appropriate design and reporting practices in future SCA/D incidence trials. This tool may also assist researchers, reviewers, journal editors, and readers in contextualizing the methodological quality of different studies with varying athlete SCA/D incidence estimates. The overarching aim of this tool is to improve our understanding of SCA/D in athletes, which carries global implications for preventative initiatives and responder policy in sport.

Because of interstudy heterogeneity in method and reporting practices, incidence estimates of SCA/D in athletes remain variable. Studies with greater methodological rigor and reporting transparency are necessary to better understand athlete SCA/D risk. Tools commonly used to assess incidence studies, such as the Joanna‐Briggs Institute critical appraisal checklist,^8^ do not include important components specific to the context of SCA/D incidence research. For example, the Joanna‐Briggs Institute checklist would not provide any assessment of inappropriate population grouping, such as age, sex, and race and ethnicity, thereby generating confounded incidence estimates with no quality scoring penalization. As such, this work addresses a substantial gap in sports cardiology research whereby no current assessment tools are well equipped for SCA/D incidence studies. Indeed, any researchers who may have attempted to perform systematic review and meta‐analysis research in this area will agree that the degree of variability between methods and reporting make any form of data pooling and interpretation of the wider literature a near‐impossible task.

The IQ‐SCA/D also has implications for the design and development of future incidence estimate trials of SCA/D, where this tool can be applied as a general framework as for guiding design and reporting practices of future research. Future studies may consider the relevant domains for study design, including the numerator and denominator quality, in their development and written contextualization of incidence estimates. Similarly, the reporting domains, including age range, sex‐specific reporting, and subgroup reporting, may serve as reference points for appropriate data reporting practices to minimize confounding and improve context of reported incidence estimates. Ultimately, the encouragement of researchers to appropriately consider these domains in the development of future incidence estimate trials of SCA/D in athletes is aimed toward improving the quality of research in this field.

The interobserver agreement analysis performed in this work varied from fair to almost perfect across the individual domains but was consistently substantial for the total summated scores and study quality categorization. The complexity and variability of many SCA/D studies certainly adds difficulty in achieving consistency throughout the scoring process; however, our evidence of agreement between observers with different levels of expertise in the area, and without any a priori familiarization or training, supports the potential for wider application of this tool.

### Limitations

Although we attempted to minimize limitations at all stages, the Delphi process carries significant inherent limitations with risks of specious consensus.^16^ Separately, because of the wide range of methods within the athlete SCA/D literature, the expert panel explicitly recognizes that there is not going to be any one set of criteria that will effectively encompass all studies and therefore the aim is to accurately capture most. This tool is specific and purposefully limited to assessing the overall incidence of SCA/D in athletes. This interpretation is important when considering some of the domains within this tool; for example, domain 4 penalizes studies that only looked at sports‐related SCA/D, even if that is the a priori aim of the work. Also, there is a significant lack of sensitivity data available to inform the domains of the tool and thus this tool relies almost entirely on expert opinion. Finally, future validation work, ideally performed externally by independent researchers, is needed.

## Conclusions

Following a Delphi process, this work presents the development and interobserver reliability of the IQ‐SCA/D tool, an international expert consensus tool for assessing the study quality of research reporting incidence of SCA/D in athletes. This tool may be implemented to assist in the methodological quality assessment of relevant studies and provide an expert‐informed framework to support and guide appropriate design and reporting practices in future SCA/D incidence trials.

## Sources of Funding

None.

## Disclosures

None.

## Supporting information

Data S1–S3
