# Supplementary material for: International Criteria for Reporting Study Quality for Sudden Cardiac Arrest/Death Tool
Source: J Am Heart Assoc. 2024 May 23;13(11):e033723. doi: 10.1161/JAHA.123.033723 (PMC11255648; doi:10.1161/JAHA.123.033723)
Supplement: Supplementary file 1 — Data S1–S3 [file JAH3-13-e033723-s001.pdf]

# Supplemental Material

## Supplemental Methods

## Data S1. Preliminary Draft Tool.

**Risk of Bias Tool for Studies on the incidence of Sudden Cardiac Arrest and Death in Athletes**

Study Design

Study design is an important feature related to quality.

2 points – Prospective
1 point - Retrospective/ Prospectively collected/Cross-sectional
0 points – Retrospective survey/registry

Numerator

The cases identified, or the numerator, needs to be accurate for a precise estimation of incidence. Most studies of SCA/D in athletes rely on media reports, registries or reviews of diagnoses on autopsy reports to identify cases for inclusion, therefore it is likely that cases will be missed, and the incidence will appear artificial low. There are few studies where there is required reporting to a central database of SCD in athletes. When evaluating the quality of an incidence study, the accuracy of the numerator should be considered.

2 points - Likely to include all cases of SCA/D
1 point - Reasonably likely to include most cases of SCA/D
0 points - Likely missed significant amount of cases

Denominator

The denominator of an incidence proportion is the number of persons at the start of an observation period. Studies of SCA/D should clearly define what population they are studying and how the group is determined. Many studies estimate participation (i.e. "there are about 8,000,000 high school athletes) which can result in either over or under-estimation of risk.

2 points - Precisely defined
1 point - Reasonably accurate
0 point - Estimate or use of multiplier

All SCA/D vs. Sports-related SCA/D

Sports-related SCA/D and SCA/D that occurs in an athlete with any activity or during any time are different but this is often not recognized. Sports-related SCA/D is typically defined as death that occurs during or within an hour of death and is a subset of all SCA/D in athletes. Sports-related SCA/D is an important metric to consider when event planning or creating emergency action plans, however, it should not be compared or conflated with SCA/D that occurs at any time.

2 points - SCA/SCD
1 point - SCD
0 points - SR-SCD or time-limited (during the school day, etc.)

Age Range

Grouping wide ranges of ages together can lead to inaccurate estimates of the incidence of SCA/D. Population based studies demonstrate a peak in SCD in those < 1 year of age followed by a relatively low rate of SCD that increases again around age 15 before rising precipitously at age 25 due to the increasing contribution of coronary artery disease. In those under 25 years old, the primary causes of SCA/D are congenital. Many studies of SCA/D group wide swaths of ages (12-40 years old) with widely varying incidence rates together. For an accurate estimation of incidence rate, it is important that age grouping reflects similar prevalence of SCA/D in that group.

2 points - Age groups generally aligned with risk (high school, college, 14-18, etc.)
1 point - Age groups include varying risk but don't include overlapping primary etiologies
0 points - Wide age range with varying risk (age groups with different predominant etiologies – congenital vs. CAD) combined

Sex-specific rates

Studies of SCA/D in athletes and non-athletes alike have consistently shown that males have a much higher rate of SCA/D than females. In general, males have 3 – 4 times higher rates of SCA/D. Combining both male and females in the same groups artificially lowers the risk for males and increases it for females. This is such an important feature that a study would score 2 points if present and 0 points if not.

2 points - Sex-specific groups
0 point – Males/females cannot be differentiated

Sub-group reporting

There may be important sub-group risks such as sport or race. This score ranges from 0 to 1.

1 point -In addition to the primary analysis, data is separately reported by sub-groups where relevant (e.g. athlete studies report risk by sport, military studies report risk by activity -basic training vs. regular duty vs. combat)

0 point - There is no sub-group data reporting

Are there other domains or elements we should consider?

**Data S2.** **D**elphi Process Expert Voting Document with Results – Round 1.

**https://forms.office.com/Pages/AnalysisPage.aspx?AnalyzerToken=eBQtaWOAxTUnENrf1h1cPiyM0HlaDWdh&id=2rIgA90iq02MIW5kS6FPE5DVwuL9WUdKvIMUoENMF3tUQlg2ODJKMEhUU1YySjhJOU9ORVRZVTZXTi4u**

**Data S3.** Delphi Process Expert Voting Document – Round 2.

**https://forms.office.com/Pages/AnalysisPage.aspx?AnalyzerToken=NMCJFa0P3KXPYpaKWEXfElikjHzshSf5&id=2rIgA90iq02MIW5kS6FPE5DVwuL9WUdKvIMUoENMF3tUNlpTVzNCM0pWWUpNUDlEM1RNS0gxQkRQWS4u**
